# Bone Morphogenetic Protein 4 Targeting Glioma Stem-Like Cells for Malignant Glioma Treatment: Latest Advances and Implications for Clinical Application

**DOI:** 10.3390/cancers12020516

**Published:** 2020-02-24

**Authors:** Sonali Nayak, Ashorne Mahenthiran, Yongyong Yang, Mark McClendon, Barbara Mania-Farnell, Charles David James, John A. Kessler, Tadanori Tomita, Shi-Yuan Cheng, Samuel I. Stupp, Guifa Xi

**Affiliations:** 1 Division of Pediatric Neurosurgery, Ann & Robert H. Lurie Children’s Hospital of Chicago, Northwestern University Feinberg School of Medicine, Chicago, IL 60611, USA; sonali.nayak@northwestern.edu (S.N.); ashornemahenthiran@gmail.com (A.M.); ttomita@luriechildrens.org (T.T.); guifa.xi@northwestern.edu (G.X.); 2 Department of Neurology, Northwestern University Feinberg School of Medicine, Chicago, IL 60611, USA; yongyong.yang@northwestern.edu (Y.Y.); jakessler@northwestern.edu (J.A.K.); shiyuan.cheng@northwestern.edu (S.-Y.C.); 3 Simpson Querrey Institute, Northwestern University, Chicago, IL 60611, USA; trospertrain@gmail.com (M.M.); s-stupp@northwestern.edu (S.I.S.); 4 Department of Biological Science, Purdue University Northwest, Hammond, IN 46323, USA; Bmania@pnw.edu; 5 Department of Neurological Surgery, Northwestern University Feinberg School of Medicine, Chicago, IL 60611; USA; Charles.james@northwestern.edu; 6 Department of Materials Science and Engineering, Department of Chemistry and Department of Biomedical Engineering, Northwestern University, Evanston, IL 60208, USA; 7 Department of Medicine, Northwestern University, Chicago, IL 60611, USA

**Keywords:** bone morphogenetic protein 4, molecular mechanism, delivery, clinical application, malignant glioma

## Abstract

Malignant gliomas are heterogeneous neoplasms. Glioma stem-like cells (GSCs) are undifferentiated and self-renewing cells that develop and maintain these tumors. These cells are the main population that resist current therapies. Genomic and epigenomic analyses has identified various molecular subtypes. Bone morphogenetic protein 4 (BMP4) reduces the number of GSCs through differentiation and induction of apoptosis, thus increasing therapeutic sensitivity. However, the short half-life of BMP4 impedes its clinical application. We previously reviewed BMP4 signaling in central nervous system development and glioma tumorigenesis and its potential as a treatment target in human gliomas. Recent advances in understanding both adult and pediatric malignant gliomas highlight critical roles of BMP4 signaling pathways in the regulation of tumor biology, and indicates its potential as a therapeutic molecule. Furthermore, significant progress has been made on synthesizing BMP4 biocompatible delivery materials, which can bind to and markedly extend BMP4 half-life. Here, we review current research associated with BMP4 in brain tumors, with an emphasis on pediatric malignant gliomas. We also summarize BMP4 delivery strategies, highlighting biocompatible BMP4 binding peptide amphiphile nanostructures as promising novel delivery platforms for treatment of these devastating tumors.

## 1. Introduction

Malignant gliomas are the most aggressive category of primary brain tumor [1]. Despite decades of research, curing these tumors remains a challenge [2]. The incidence of malignant gliomas differs by age. In adults (≥19 years), the average overall annual incidence is 8.82 per 100,000. In children (<19 years), malignant gliomas include anaplastic astrocytoma, glioblastoma and diffuse intrinsic pontine gliomas (DIPGs), with an average annual incidence of 3.48 per 100,000 [1]. Regardless of age, patients with these devastating tumors have a poor clinical prognosis [3,4]. Radical surgical resection followed by adjuvant radiotherapy and/or chemotherapy are standard treatments for these tumors, however, tumor recurrence occurs in nearly all instances, primarily due to intrinsic or acquired resistance to routinely used therapies [5]. Identifying novel therapeutic approaches to improve survival in patients with these malignancies is imperative.

Data from the Central Brain Tumor Registry of the United States (CBTRUS) reveals differences between adult and pediatric patients including tumor incidence and location [1]. Genomic and epigenomic analyses have also shown significant differences between adult and pediatric tumors [6,7]. In adult high-grade gliomas (aHGGs), epidermal growth factor receptor (EGFR) is a commonly altered receptor tyrosine kinase (RTK) and mosaic expression of platelet-derived growth factor receptor-α (PDGFRA), platelet-derived growth factor α (PDGFA), fibroblast growth factor receptor 1(FGFR1), fibroblast growth factor 1 (FGF1), NOTCH2, JAG1 (Jagged Canonical Notch Ligand 1) are common. Additionally, IDH1 mutations have been identified in glioblastomas developed from WHO grade II/III astrocytomas or oligodendrogliomas [8,9,10,11]. In pediatric high-grade gliomas (pHGGs), PDGFRA is a more common RTK alteration and MYC and MYCN are frequently amplified [12]. Furthermore, multiple hotspot histone mutations have been identified in pHGGs, but are rare in aHGG. These histone mutations vary further between different pHGG tumor types. For instance, mutations in H3, family 3A (H3F3A) and histone cluster 1, H3b (HIST1H3B), occur at lysine 27 (K27M) in ~80% of DIPGs [13,14], a subset of pHGGs arising from the brainstem. Mutations on histone H3G34 (G34V/R) are present in ~38% of hemispheric pHGGs [12]. In addition to histone mutations, TP53 and activin receptor type 1 (ACVR1, also known as ALK2) mutations are frequent in DIPG [15,16,17], and chimeric fusions involving the kinase domain of neurotrophic tyrosine kinase receptors are present in ~40% of hemispheric pHGG [13]. 

Regardless of the aforementioned molecular differences between aHGGs and pHGGs, a small population of glioma stem-like cells (GSCs) are considered a driving force for tumor growth and recurrence, and tumor heterogeneity [18,19,20,21]. GSCs can initiate tumors that reproduce the parental tumors’ cellular heterogeneity. GSCs also resist the cytotoxic effects of radiation and chemotherapy [22,23,24,25,26]. These findings indicate that GSCs may be critical therapeutic targets. 

Bone morphogenetic protein 4 (BMP4) can abolish cancer stem cell populations in human cancers [27,28,29,30,31,32], including in malignant gliomas [33,34,35,36,37]. In a current phase I clinical trial (NCT02869243) human recombinant BMP4 is being administered through intratumoral and interstitial convection-enhanced delivery (CED) for adult glioblastoma treatment (https://clinicaltrials.gov/ct2/show/NCT02869243). BMP4 signal pathways appear to play critical roles in the regulation of malignant glioma tumor biology, further suggesting that it is a promising therapeutic molecule. However, to fully elucidate BMP4 therapeutic potential, differential roles of BMP4 in tumor molecular subgroups should be examined. In addition, to take advantage of this potential, novel biocompatible materials for effective BMP4 binding and delivery are being synthesized. Preliminary unpublished results from our laboratory showed that an innovative biocompatible peptide amphiphile nanostructure binds BMP4 and markedly extends its half-life, an important factor for its clinical utility [38]. In this review, we have discussed the recent discoveries elucidating the role of BMP4 signal pathways in malignant gliomas and reviewed innovative biocompatible materials for BMP4 delivery and their prospects for clinical applications.

## 2. BMP4 Signal Pathways and Glioma Biology 

BMP4 is a member of the TGF-β family. BMP4 signal pathways are critical in early embryonic development, central nervous system (CNS) formation and development through regulation of stemness and differentiation of neural stem cells (NSCs) [39,40]. In the subventricular zone of the adult brain, BMP4 promotes NSC differentiation into astrocytes [41]. BMP4 directly binds to BMPR1A, BMPR1B, and BMPRII, resulting in phosphorylation of cytosolic Smad (mothers against decapentaplegic homolog) proteins. Smad proteins translocate to the cell nucleus, where they bring about Smad-mediated gene expression as well as activation of MAPK (mitogen-activated protein kinase) signaling as described [42]. Increasing evidence indicates that BMP4 signaling pathways are relevant to human gliomas. However, the role of BMP4 signaling pathways varies between aHGGs and pHGGs, due to differences in the molecular background. For example, a mutation in ACVR1, a member of the BMPRI family, is more frequent in pHGGs, compared to aHGGs [12,15,16,17,37,43,44]. In light of the molecular background differences, BMP4 action needs to be interpreted with respect to the distinct features of each tumor group.

## 3. BMP4 Signaling in Adult High-Grade Gliomas

**Figure 1:**
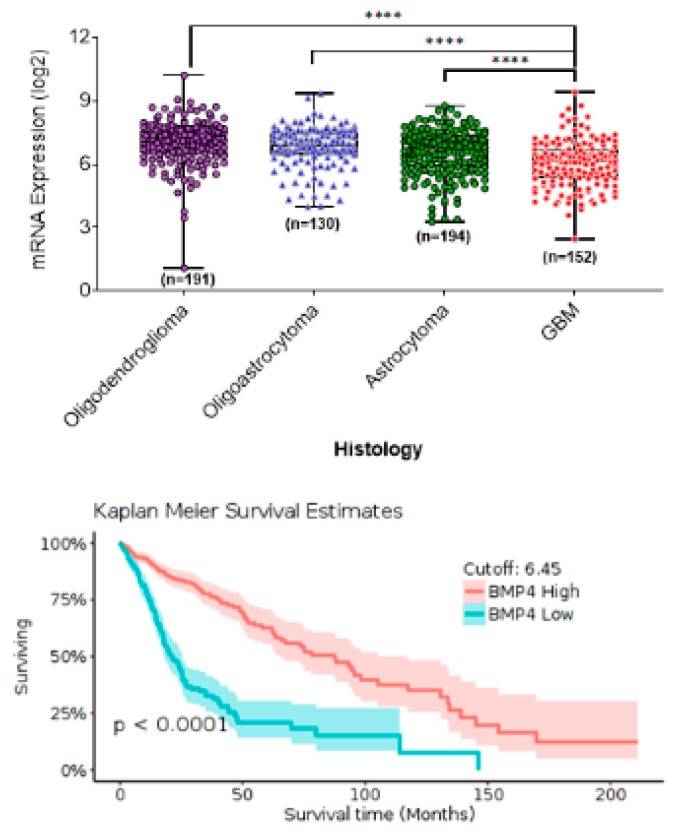
In silico data analysis from The Cancer Genome Analysis (TCGA) using the Gliovis online portal shows BMP4 mRNA expression associated with tumor category based on histology (**top panel**) and patient survival in adult gliomas (**bottom panel**).

HGGs are the most common solid CNS adult tumors. We analyzed the Data from The Cancer Genome Analysis (TCGA) using the GlioVis data portal for visualization and analysis of brain tumor expression datasets [45] (http://gliovis.bioinfo.cnio.es/). The results showed that low-grade gliomas (LGG) express higher BMP4 levels and exhibit lower mortality rates than HGGs that express lower levels of BMP4 (Figure 1). These results were consistent over multiple data sources [46,47], and suggest that BMP4 can be a robust prognostic marker for adult gliomas. The results further suggest that therapeutic targeting of BMP4 may be an effective strategy for treating aHGG. 

GSCs are considered a source for tumors and these cells are resistant to radiation and chemotherapy [48,49]. One strategy to improve treatment outcomes for aHGG is to target GSCs to improve tumor response to conventional therapies. Another strategy is to induce GSC differentiation, resulting in a reduction of the tumorigenic cell population [50]. Treatment with BMPs, including BMP4 provides an approach for inducing GSC differentiation. GSCs express BMP receptors, and have a functional BMP4 signal pathway. The addition of exogenous BMP4 to GSCs enhances SMAD phosphorylation and reduces GSC proliferation [41,51]. Furthermore, in response to BMP4, CD133, a GSC marker, decreases, whereas GFAP, a marker for differentiated astrocytes, increases. Treatment with exogenous BMP4 also decreases GSC tumorigenicity in vivo [51] and reduces tumor cell proliferation [52]. These results, in total, suggest that BMP4 promotes GSC differentiation, and may prove useful in treating HGGs [53]. BMP4 also reduces multidrug resistance in glioma cells and suppresses glioblastoma invasiveness. Multidrug resistance is reduced through the inhibition of B-cell lymphoma 2 (BCL-2) and glial cell derived neurotrophic factor (GDNF), while invasiveness is reduced through increased E-cadherin and claudin expression [33]. 

**Figure 2:**
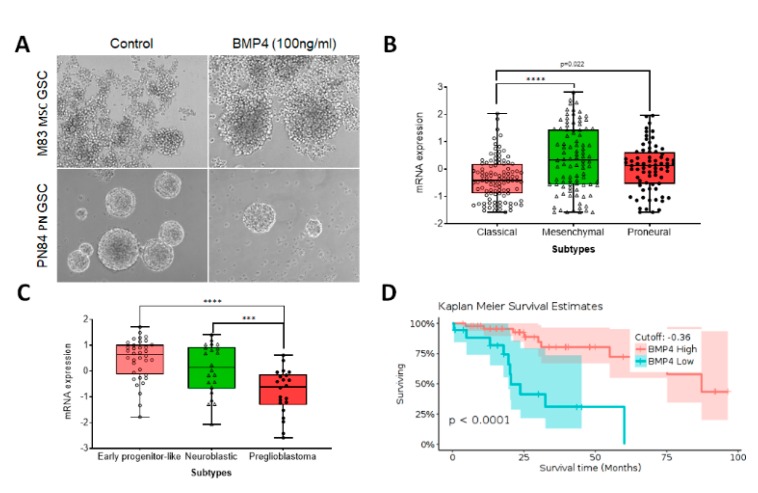
BMP4 expression in adult glioblastoma subtypes, associated with patient outcome. (**A**) Representative images show that mesenchymal stem cell (MSC) subtype M83 glioma stem-like cells (GSCs) and proneural (PN) PN84 GSCs respond differentially to BMP4 treatment for 96 h, with only PN84 GSCs showing decreased proliferation. (**B**–**D**) In silico TCGA data analysis with Gliovis indicates that: BMP4 levels vary in adult glioblastoma subtypes (**B**); BMP4 expression varies in isocitrate dehydrogenase 1 (IDH1) mutant early progenitor-like, neuroblastic and preglioblastoma cells (**C**) and BMP4 level is associated with survival in patients with IDH1 mutant glioblastoma (**D**).

With advances in biotechnology, including integrative application of high-throughput sequencing such as single cell RNA-seq (scRNA-seq), 450K DNA methylation profiling, high-throughput m^6^A-seq, and whole-genomic sequencing (WGS), it is possible to obtain precise molecular signatures, and identify the diverse genetic and epigenetic programs that drive cancers such as gliomas. For example, scRNA-seq reveals proneural, classic and mesenchymal GSC subtypes within individual tumors, thus demonstrating intratumoral cellular heterogeneity [10,54,55]. Preliminary results from our laboratory have indicated that these cell subtypes respond differently to BMP4 (Figure 2A). Proliferation of mesenchymal subtype GSCs does not decrease following treatment with 100ng/ml BMP4 for 4 days. In comparison, proliferation of proneural subtype GCSs does decrease under these conditions. This may reflect different levels of endogenous BMP4 expression (Figure 2B). For instance, the mesenchymal glioblastoma subtype expresses higher levels of BMP4 than proneural and classic GSC subtypes. The pre-glioblastoma subtype within isocitrate dehydrogenase 1 (IDH1) mutant gliomas express low BMP4 (Figure 2C) [56], in comparison to early progenitor-like and neuroblastic subtypes, and is associated with a poor patient prognosis (Figure 2D) [54,57]. 

## 4. BMP4 Signaling in Pediatric High-Grade Gliomas 

Pediatric brain tumors are distinct from their adult counterparts in terms of epidemiology, cellular origins, response to cytotoxic and radiation therapy and clinical outcomes. Recent wide-spread genome-wide profiling that has been applied to pediatric brain tumors has provided full characterization at the molecular genetic level. These large-scale analyses have revealed distinct tumor driving events, gene expression profiles, mutation targets and mutation frequencies [7]. Accordingly, BMP4 involvement in pHGG biology warrants its own examination. 

**Figure 3:**
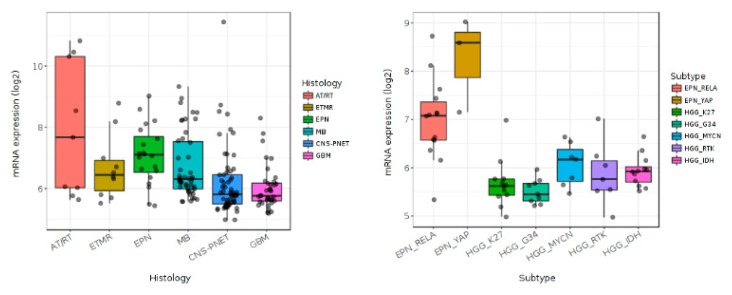
In silico analysis of pediatric brain tumor datasets (GEO: GSE73038) with Gliovis showing differential expression of BMP4 in pediatric central nervous system tumors (**left panel**) and pediatric high grade gliomas (right panel).

In silico analysis of data from dataset GEO: GSE73038 ([58] shows that BMP4 is differentially expressed among histopathologically-defined pediatric CNS brain tumors (Figure 3, left panel), including in pHGGs (Figure 3, right panel). DIPGs, pHGGs arising in the brainstem, are characterized by an H3K27M mutation in either histone H3.1 or H3.3. H3.3 K27M mutations are also present in other pHGGs from midline regions, including from areas such as the thalamus, cerebellum and spine [59]. While H3.1 K27M mutations are restricted to DIPG [60], they usually occur in conjunction with abnormal signaling pathway activity including pathways associated with BMP4 [14,15,16,29,61]. Recurrent somatic mutations involving ACVR1 have also been discovered in DIPGs [13,15,16]. Interestingly, gain-of-function mutations in ACVR1 appear to be restricted to DIPGs with an H3.1 K27M of the HIST1H3B gene, and are not present in DIPGs with H3.1 K27M mutation of the H3F3A gene. DIPGs harboring ACVR1 mutations exhibit hyperactivation of BMP-ACVR1 signaling, which results in elevation of phosphorylated SMAD1/5/9 and increased expression of BMP downstream response genes [16]. DIPG patients whose tumor harbors an ACVR1 mutation show improved survival [13,17]. Therapeutic targeting of AVCR1 has beneficial anti-tumor effects in preclinical DIPG models [43]. However, the targeting effect is mutation domain dependent [43,44].

pHGGs also display other histone mutations, for instance the histone H3G34 (G34V/R) mutation is present in hemispheric pHGGs [12]. Preliminary unpublished results from our laboratory showed increased phosphorylated SMAD1/5/9 and decreased multidrug resistance gene 1 (MDR1) expression, in pediatric glioblastoma KNS42 cells harboring an H3F3A G34V mutation, following BMP4 treatment. Decreasing MDR1 increases tumor cell sensitivity to cytotoxic therapies. Thus, the result with KNS42 cells indicates that, in addition to ACVR1 mutated H3.1 K27M DIPGs, other pHGGs may benefit from BMP4 targeted therapy. 

## 5. BMP4 Delivery Methods for Glioma Treatment

**Figure 4:**
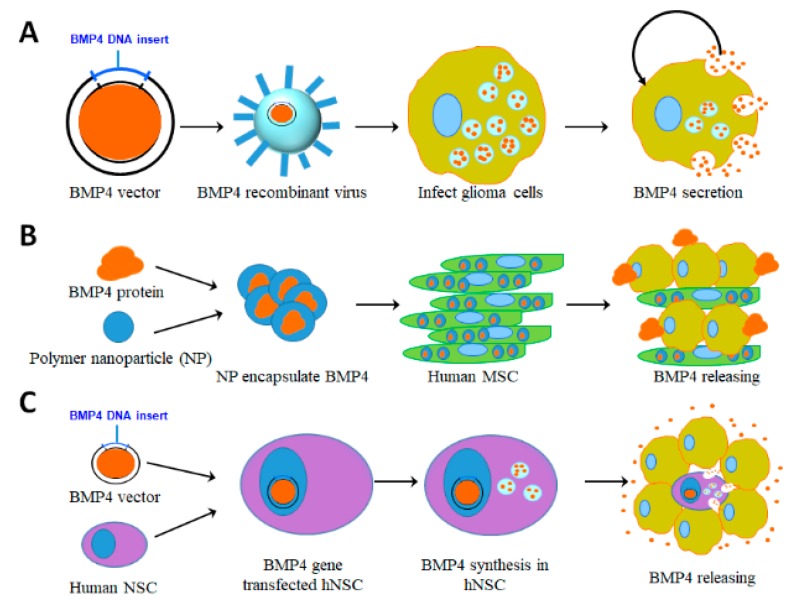
Representative illustrations indicate BMP4 delivery methods. (**A**) Viral vector based delivery. BMP4 plasmids are transduced into oncolytic virus to infect glioblastoma cells, which consequently induces apoptosis and differentiation to improve the therapeutic outcome. (**B**) Human mesenchymal stem cell (hMSC) based delivery. BMP4 is encapsulated into polymer nanoparticles (NP) and transfected into human adipose MSCs, which sustainably release BMP4 to target glioblastoma cells. (**C**) BMP4 plasmids are transfected into human neural stem cells (NSCs). When these NSCs are co-cultured with glioblastoma cells or injected into glioblastomas, BMP4 is produced and released to target tumor cells.

**Figure 5:**
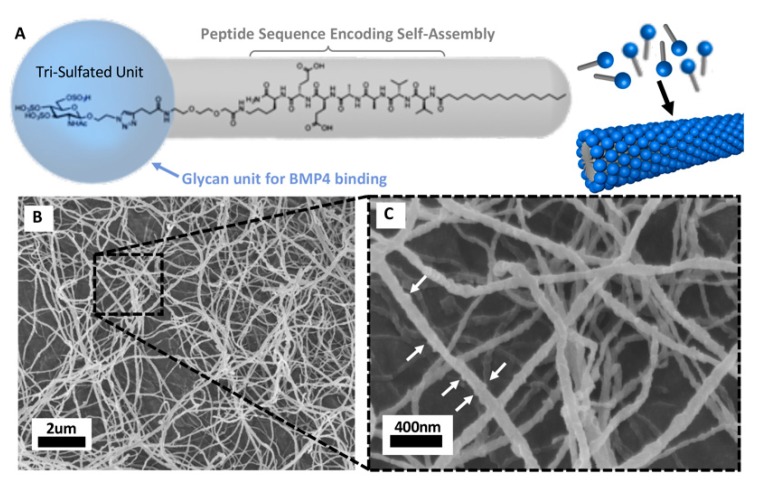
Peptide amphiphile (PA) nanostructures are novel biocompatible materials for BMP4 delivery. (**A**) Molecular structure of a glycosylated PA molecule and schematic of the self-assembled glycosylated PA nanofiber. The grey molecular region guides the self-assembly process into nanofibers, the blue molecular region contains the tri-sulfated monosaccharide responsible for protein binding actions. (**B**) SEM of glycosylated PA nanofiber bundles after exposure to blood proteins for 5 min. (**C**) Higher magnification of nanofibers with white arrows indicating a rigid surface texture resulting from proteins binding to the fiber surface. (Figures provided by Dr. Samuel Stupp with permission for publication).

The BMP signaling pathway is a potential therapeutic target for treating gliomas. Therapeutic applications of BMP4 for both adult and pediatric HGGs are based on its ability to induce differentiation and apoptosis of GSCs and thus reduce this cell population. The mechanism for BMP differentiation therapy involves driving GSCs into a post-mitotic state that limits tumor growth. However, there are obstacles that must be overcome relative to BMP4 clinical treatment of malignant gliomas via differentiation therapy [62,63]. For instance, autocrine BMP4 enhances tumor aggressiveness in IDH1 mutant gliomas [64]. It is possible that only certain subsets of GSCs, based on molecular characteristics, are targetable in response to high doses of BMP4. Some cell subsets may show incomplete cell-cycle arrest and/or tumor cell retention of growth-promoting DNA methylation patterns [63]. Further investigation of cell molecular characteristics and differentiation needs to be done to help overcome these obstacles. Another limiting factor for clinical application of BMP4 is its short half-life [65,66,67]. One strategy for overcoming this limitation is the delivery of large doses of BMP4 via polymer beads [51]. Other delivery systems have been designed to overcome the short half-life of BMP4 and improve its biomedical effects, including recent advances in the synthesis of biocompatible BMP4 binding materials. The following discussion reviews delivery systems (Figure 4) and our innovative peptide amphiphile nanostructures as an innovative BMP4 delivery platform (Figure 5). 

### 5.1. Viral Vector Based Delivery

Viral vectors have been used for high efficiency gene delivery, including for the production of BMP4 in gliomas. An oncolytic vaccinia virus (VACV) expressing BMP4 was delivered both in vitro to primary glioma cultures and in vivo intracranially to xenograft gliomas (Figure 4A). The results of the in vitro study showed cytotoxic activity against GSCs and the in vivo study improved survival rates in treated mice and reduced recurrence of glioma following VACV infection [68]. VACVs, however, are associated with risks including neurodegeneration and demyelination, which limits their clinical application for expressing BMP4 in gliomas [69].

### 5.2. Human Adipose-Derived Mesenchymal Stem Cell (hAMSC) Based Delivery

Because of their high glioma tropism, human adipose-derived mesenchymal stem cells (hAMSCs) have been touted as a potential therapeutic delivery vehicle for glioma treatment. Though originally derived from bone marrow, large amounts of MSCs can be isolated from adipose tissue, with cells from either source relatively equivalent in treatment efficacy [70]. Furthermore, hAMSCs can be altered with nanoparticles to be more effective than conventional polymers in delivering BMP4 [70]. Nanoparticle-engineered hAMSCs expressing BMP4 cross the blood brain barrier, migrate to and penetrate intracranial tumors, and extend survival. In vivo and in vitro studies showed that hAMSC-BMP4 decreased migration and proliferation of GSCs while promoting differentiation (Figure 4B). Additionally, mice bearing murine GBM experienced improved survival after treatment with hAMSC-BMP4. Significantly, in vivo, hAMSCs maintain their multipotency and hAMSC malignant transformation has not been observed, despite exposure to the GBM microenvironment [71]. However, with the application of tumor growth factors, hAMSCs can transform into fibroblasts and potentially contribute to tumor expansion [71,72]. Further, hAMSCs stop proliferating in vivo after a few days, so that the effect of BMP4 from hAMSC production is limited [73].

### 5.3. Human Neural Stem Cell (hNSC) Based Delivery

To address deficiencies in the distribution of “free” oncolytic vectors, the use of virally transduced human neural stem cells (hNSCs) has been proposed to treat gliomas. These cells would deliver conditionally replicating adenovirus (CRAd) (Figure 4C). NSCs have shown an intrinsic migratory capacity towards brain tumors, though the mechanisms of this tropism are poorly understood [70,74]. Harnessing the homing ability of hNSCs in conjugation with BMP4 expression inhibits GSC growth both in vivo and in vitro, likely via the Smad signaling pathway. In vivo, hNSC-BMP4 treatment is effective in promoting GSC differentiation and apoptosis in xenograft gliomas, and improves the survival of mice bearing these tumors [74]. 

### 5.4. Biocompatible Nanomaterial Based Delivery

Self-assembling materials such as peptide amphiphiles (PAs) have been a focus of medical applications over the past two decades. PAs can be designed to self-assemble in cylindrical nanostructures that resemble the structural characteristics of native extracellular matrix (ECM) fibers. The molecular design of PAs allows for the incorporation of bioactive signals that will be displayed on the surface of the self-assembled nanofibers creating opportunities for exciting novel therapies with broad potential impact in regenerative medicine and cancer. Recently, Srikanth et al. [75] reported that PA nanofibers displaying an IKVAV peptide signal could be used to treat GSCs. They showed that this specific PA potentially increases immobilized β1-integrin at the GSC membrane, activating integrin-linked kinase while inhibiting focal adhesion kinase (FAK), which consequently induces apoptosis in GSCs. PA nanofibers can also be designed to display binding peptide sequences allowing the nanofibers to bind and deliver specific proteins, nucleic acids, drugs and cells [76]. For example, PA nanofibers as a delivery mechanism have been investigated to deliver BMP2. This particular PA nanofiber displays a peptide sequence found through phage-display techniques with an affinity for BMP2. The use of this binding nanofiber led to more efficient delivery and protein activity that resulted in a ten-fold dose reduction of BMP2 required for successful spinal fusion in a rat model [77]. More recently, Lee et al. [38] synthesized a novel sulfated glycopeptide nanostructure that has a binding affinity for multiple proteins including BMP4 (Figure 5). Most importantly, these PA nanostructures are biocompatible, thus they do not cause side effects while providing more efficient delivery to increase therapeutic benefit.

## 6. Future Prospects

Here, we have summarized recent BMP4 associated progress in aHGG and pHGG. BMP4 treatment could be a valuable adjunct to conventional therapies for these devastating tumors. However, BMP4 mediated differentiation therapies must be used in a patient-specific context since a subset of gliomas do not differentiate in response to BMP4 [21,78,79]. To better predict therapeutic value, the roles of BMP4 in subsets of aHGGs and pHGGs with specific molecular signatures should be further examined. The means delivering BMP4 is also a key factor. We described current BMP4 delivery strategies and proposed that biocompatible nanocarriers could be a novel highly efficient delivery platform. Further studies need to develop PA nanostructures for brain tumor treatment via systemic administration. These nanostructures must be designed to have high BMP4 affinity and to cross the blood-brain barrier. We hope advanced nanotechnology based on self-assembling peptides will enhance BMP4 delivery efficacy and lead to new therapeutic options that, in combination with conventional cytotoxic and/or radiation therapy, will improve outcomes for patients with HGGs.
